# Effect of whole-body vibration training on bone mineral density in older adults: a systematic review and meta-analysis

**DOI:** 10.7717/peerj.19230

**Published:** 2025-05-16

**Authors:** Danilo A. Massini, Tiago A. F. Almeida, Anderson G. Macedo, André B. Peres, Víctor Hernández-Beltrán, José M. Gamonales, Mário C. Espada, Cassiano M. Neiva, Dalton M. Pessôa Filho

**Affiliations:** 1 São Paulo State University (UNESP), Bauru, SP, São Paulo, Brazil; 2 São Paulo State University (UNESP), Rio Claro, SP, São Paulo, Brazil; 3 Federal University of Alfenas (UNIFAL), Alfenas, MG, Minas Gerais, Brazil; 4 São Paulo Federal Institute of Education, Science and Technology (IFSP), Piracicaba, SP, São Paulo, Brazil; 5 Training Optimization and Sports Performance Research Group (GOERD), Cáceres, Cáceres, Spain; 6 Faculty of Education and Psychology, University of Extremadura, Badajoz, Badajoz, Spain; 7 Universidad a Distancia de Madrid, Collado Villalba, Madrid, Madrid, Spain; 8 Instituto Politécnico de Setúbal, Setúbal, Portugal; 9 Life Quality Research Centre (CIEQV-Setúbal), Setúbal, Setúbal, Portugal; 10 Centre for the Study of Human Performance (CIPER), Universidade de Lisboa, Lisboa, Portugal; 11 Comprehensive Health Research Centre (CHRC), Universidade de Évora, Évora, Portugal; 12 Sport Physical Activity and Health Research & Innovation Center, Santarém, Santarém, Portugal

**Keywords:** Aging, Bone mineral density, Vibration stimuli, Training plans

## Abstract

**Background:**

Whole-body vibration (WBV) aims to increase bone mineral density (BMD) using vertical mechanical accelerations from the plantar surface of the feet through the muscles and bones. A vibration platform is used for this purpose. This systematic review (PROSPERO—CRD 42023395390) analysed the effects of WBV training on BMD at anatomical sites most affected by osteoporotic fractures in older adults.

**Methodology:**

Systematic searches were conducted in the databases. Randomized controlled studies quantifying aerial BMD (aBMD) using the dual-energy X-ray absorptiometry method before and after WBV training in adults aged 55 and older were included. Independent reviewers performed methodological quality analysis (TESTEX) and assessed the risk of bias, and the GRADE scale determined the certainty of evidence in the results of the selected studies. The aBMD values from anatomical sites in the femoral neck, total proximal femur, and lumbar spine from WBV training protocols were included in the meta-analysis. The forest plot was generated using the random-effects model, and the effect size was measured by Hedges’ *g*.

**Results:**

Seven studies involving 202 participants were included, with TESTEX = 12.6 (excellent quality) and risk of bias (43% low risk, and 57% some concerns), demonstrating with low heterogeneity, a significant effect of WBV training on total femur aBMD (*g* = 0.28 (small), *p* = 0.04). However, in spite of the low heterogeneity, the femoral neck (*g* = 0.15 (trivial), *p* = 0.19) and lumbar spine (*g* = 0.13 (trivial), *p* = 0.31) regions did not show a significant effect with WBV training.

**Conclusions:**

The results showed with low certainly evidence that WBV training had a statistically significant effect on total femur aBMD but not on femoral neck and lumbar spine.

## Introduction

The aging process involves morphological and functional declines related to both biological, such as neuromuscular activation and muscle mass reduction (Fischer et al., 2019; Choe, Jeong & Kim, 2020), and lifestyle factors stemming from sedentary behaviour and poor dietary habits (Tieland, Trouwborst & Clark, 2018; Massini et al., 2022). Consequently, these factors also affect the skeletal system, as evidenced by reductions in bone mineral density (BMD) (Gomez-Cabello et al., 2012; Abazović, Paušić & Kovačević, 2015; Mohammad Rahimi et al., 2020; Massini et al., 2022). This bone reduction is more pronounced in women, with an annual decrease of around 5% in the first years after menopause, followed by a yearly loss of 2% to 3%, and in men with reductions of 1% to 2% in old age (Gomez-Cabello et al., 2012). BMD reflects the amount of bone mineral mass within the bone region of interest (ROI), therefore this has been used as an index for the risk of developing pathologies and injuries such as osteopenia and osteoporosis (Abazović, Paušić & Kovačević, 2015; Zha et al., 2012; Mohammad Rahimi et al., 2020; Zamoscinska, Faber & Busch, 2020). Clinically, osteoporosis is a silent disease characterized by increased bone resorption and an inadequate compensatory balance in forming new bone tissue (Gomez-Cabello et al., 2012; Abazović, Paušić & Kovačević, 2015; Massini et al., 2022). Fractures are more frequently observed in the femur, spine, and hip regions, with a global annual rate of 10 million fractures (Camacho-Cardenosa et al., 2019).

Physical training has been considered a non-pharmacological alternative for preventing and treating osteopenia and osteoporosis (Liu, Brummel-Smith & Ilich, 2011; Abazović, Paušić & Kovačević, 2015; Marín-Cascales et al., 2018; Fernandez et al., 2022; Hejazi, Askari & Hofmeister, 2022). Thus, various exercise modalities, such as aerobic (Liu, Brummel-Smith & Ilich, 2011; Beavers et al., 2017; Mohammad Rahimi et al., 2020) and resistance training (Bemben et al., 2010; Massini et al., 2022), either planned alone or combined, have been investigated and the effect on BMD maintenance or enhancement being evidenced (Villareal et al., 2017; Mohammad Rahimi et al., 2020; Massini et al., 2022). Aside from the benefits of physical training, whole-body vibration (WBV) training has also emerged as an alternative for increasing BMD (Verschueren et al., 2004; Beck & Norling, 2010; Slatkovska et al., 2010; Santin-Medeiros et al., 2015; Marín-Cascales et al., 2018; Camacho-Cardenosa et al., 2019; Mohammad Rahimi et al., 2020; Fernandez et al., 2022). However, the physiological effects of WBV training are still not fully understood and its efficacy for safe clinical application requires further research (Moreira-Marconi et al., 2019). WBV training involves placing an individual in a standing or squatting position on a vibrating platform (*e.g*., vertical or side-alternating) (Slatkovska et al., 2010; van Heuvelen et al., 2021; Harijanto et al., 2022), where vertical accelerations in relation to the ground, starting from the plantar surface of the feet, transmit mechanical vibration through the muscles and bones supporting the body mass (Slatkovska et al., 2010; Abazović, Paušić & Kovačević, 2015). The intensity of WBV is defined by its frequency (hertz, Hz), magnitude expressed as vertical acceleration (*i.e*., g = 9.81 m/s^2^ acceleration due to gravity), oscillations (1–10 mm), and planes (sagittal, frontal, and transversal) (Slatkovska et al., 2010; Fernandez et al., 2022; van Heuvelen et al., 2021; Harijanto et al., 2022). Training recommendations suggest using frequencies between 20 and 40 Hz and amplitudes between 2.0 and 5.0 mm, with daily sessions lasting up to 30 min, conducted three times a week (Abazović, Paušić & Kovačević, 2015; Harijanto et al., 2022).

The hypothetical mechanism underpinning the WBV training effect is the osteogenic stimuli with muscle activation (*i.e*., tensional stimuli) (Marín-Cascales et al., 2018), resulting in the mechanotransduction of vibration-induced stresses in the bone through the activation of Piezo 1 and 2 ion channels (Slatkovska et al., 2010; Moreira-Marconi et al., 2016; Bemben et al., 2018; Mohammad Rahimi et al., 2020; Cheng et al., 2021). This mechanical signalization further stimulates physiological responses (*e.g*., hormonal releasing) linked to bone metabolism modulation, hence favouring the increase in mineral content and density (Sá-Caputo et al., 2023). Another hypothesis is that mechanical forces applied to the bone tissue induce interstitial fluid movement along the canaliculi and lacunae of osteocytes, causing cellular-level shear stress and deformations of the osteocyte plasma membrane (Slatkovska et al., 2010; Dionello et al., 2016; Mohammad Rahimi et al., 2020). These changes induce bone to enhance the mineral remodelling process, stimulating bone formation (Liu, Brummel-Smith & Ilich, 2011; Abazović, Paušić & Kovačević, 2015; Dionello et al., 2016; Mohammad Rahimi et al., 2020). However, there is still no consensus on the effect of WBV training on BMD in different body regions (Abazović, Paušić & Kovačević, 2015; Zha et al., 2012; Marín-Cascales et al., 2018; Fernandez et al., 2022; de Oliveira et al., 2023), which might be accounted to the different variables (*i.e*., frequency, intensity, amplitude) of training planned for the protocols available in the literature (Abazović, Paušić & Kovačević, 2015; Zha et al., 2012; Camacho-Cardenosa et al., 2019; Wuestefeld et al., 2020; Fernandez et al., 2022; Harijanto et al., 2022) and participant characteristics (*i.e*., age, sex, training status) (Slatkovska et al., 2010; van Heuvelen et al., 2021).

In light of these miscellaneous protocols of WBV and their effects on BMD, some systematic reviews and meta-analyses aimed to discern the effect of WBV training on whole-body and regional BMD (Bemben et al., 2018; Cheng et al., 2021; de Oliveira et al., 2023). However, these investigations have methodological limitations related to confounding factors in their results (Santin-Medeiros et al., 2015; Harijanto et al., 2022), as they include studies with the combination of (i) WBV with other types of training (resistance or aerobic) (Dionello et al., 2016); (ii) treatments with dietary supplements (*e.g*., vitamins and minerals) (Slatkovska et al., 2010; Dionello et al., 2016; Marín-Cascales et al., 2018) and/or osteogenic drugs (*e.g*., medications and hormones) (Harijanto et al., 2022); and (iii) quantification of BMD using different methods (*e.g*., dual-energy X-ray absorptiometry (DXA) and computed tomography) (Dionello et al., 2016). Clearly, this is a limitation to the observation that WBV training affects bone tissue (Slatkovska et al., 2010; Moreira-Marconi et al., 2016; Harijanto et al., 2022), justifying the need for further studies to eliminate these biases from the results (Harijanto et al., 2022; Massini et al., 2022). Therefore, the current systematic review and meta-analysis aimed to discern the effect of WBV training (*per se*) on anatomical sites, constraining the evidence of the most vulnerable sites to osteoporotic fractures in older adults. Therefore, this study will contribute to providing enough evidence on the practice of WBV as a non-pharmacological rehabilitation method that is safe and cost-effective, as has been speculated by previous studies (Slatkovska et al., 2010; Fischer et al., 2019).

## Survey methodology

This systematic review and meta-analysis followed the recommendations outlined in the Cochrane Handbook for Systematic Reviews of Interventions (version 5.1.0), and its writing adhered to the PRISMA (Preferred Reporting Items for Systematic Reviews and Meta-Analyses) checklist (Page et al., 2021) (see Supplemental File). The study was registered in the International Prospective Register of Systematic Reviews (PROSPERO—CRD 42023395390) in February 2023.

High-sensitivity searches, which means not constraining the search to a given period of time and nor to a given language of publication, were conducted in the Embase, ESPORTDiscuss, LILACS, PEDro, PubMed, and SciELO electronic databases (see Supplemental File), covering studies published until December 27, 2024. The search used the Population, Intervention, Comparator, and Outcome (PICO) descriptors, as follows: Population: “*older adults*” OR “e*lderly*” OR “*aging*”; Intervention: “*whole body vibration*” OR “*vibration platform*” OR “*vibratory exercise*”; Comparator: pre- *vs*. post-training difference in BMD as a result of a WBV training program (comparison with a control group was not performed due to studies using different types of exercises, *e.g*., impact, resistance, or aerobic exercise, or not engaging in exercise, generating a confounding bias in effect size estimation) (Fischer et al., 2019; Dolan et al., 2020; Massini et al., 2022); Outcome: “*bone mineral density*” OR “*bone mineral content*” OR “*bone metabolism*” OR “*bone mass*”. The search strategy underwent peer review by an information scientist using the Peer Review of Electronic Search Strategies (PRESS) form (McGowan et al., 2016). The reliability of the search strategy was confirmed by referencing the study by Bemben et al. (2010).

Manual searches were conducted in the references of eligible articles and their citations in the PubMed, Scopus, and Google Scholar databases to add other relevant titles. Additionally, attempts were made to email the authors of the selected articles to request any missing relevant information. Two authors (DAM and ABP) conducted the searches to avoid any selection bias. After completing the searches, the authors compared the lists of included and excluded studies using the Rayyan online tool (Ouzzani et al., 2016). The discrepancies were analysed through discussion and agreement with a third author (DMPF).

### Article selection criteria

Studies that provided quantification of aerial BMD (aBMD) were included. The inclusion criteria were as follows: (i) randomized controlled studies conducted in humans aged 55 years and older; (ii) studies that quantified the aBMD of anatomical sites or body regions that present a high incidence of osteoporotic fractures (*e.g*., femoral, lumbar spine and total femur) using only DXA to consider that the measurements be a standard reference for the population, and therefore comparable (Massini et al., 2022), and not adding confounding factors with other methods (Santin-Medeiros et al., 2015; Harijanto et al., 2022); and (iii) peer-reviewed studies. In order to avoid miscellaneous definition of bone site selections, the terms femoral neck and total proximal femur provide information for a given site or region of the femur by selecting one-side of hip analysis in the DXA scan. Despite proximal femur analysis in DXA comprising the neck, trochanteric, and intertrochanteric sites, the femoral neck response to WBV training was shown separately. In turn, the lumbar spine scan included the ROI between L1 to L5. The exclusion criteria were as follows: (i) studies conducted in clinical populations that limit training protocols (cardiovascular and orthopaedic diseases) or interfere with bone metabolism (*e.g*., diabetes, obesity) (Massini et al., 2022); (ii) studies that combined WBV with other training protocols (resistance, aerobic, and impact exercises); (iii) studies administering dietary supplements or osteogenic drugs; (iv) case studies, literature reviews (systematic review and meta-analysis); and (v) studies with low methodological quality.

### Data extraction

Two authors (DAM and AGM) extracted data using a pre-pilot spreadsheet which was independently verified by a third author (TAFA) from the review team. When data were presented only in graphs, WebPlotDigitizer software (Version 4.6, WebPlotDigitizer; Pacifica, San Diego, CA, USA) was used to extract the data (Drevon, Fursa & Malcolm, 2017). The following data were extracted: (i) authors’ names; (ii) year of publication; (iii) characteristics of the population (sample size, sex, age, height, and body mass); (iv) WBV training protocol; and (v) pre- and post-training BMD.

### Methodological quality assessment and risk of bias

Two independent authors (DAM and TAFA) conducted the assessment, and discrepancies were analysed by a third author (AGM) using the “Tool for the Assessment of Study Quality and Reporting in Exercise” (TESTEX) checklist (Smart et al., 2015). The checklist assigns one point if the criterion is met and zero otherwise. It comprises two sections related to quality (items 1–5) and study reporting (items 6–12), with criteria 6 and 8 designed by three and two sub-criteria each one (respectively), amounting to 15 points (see detailed information in Table 1). Based on summarized scores, studies were classified as “excellent quality” (12–15 points), “good quality” (9–11 points), “fair quality” (6–8 points), or “poor quality” (<6 points) (Nunes et al., 2020) (Table 1).

**Table 1:** Methodological quality assessment using the TESTEX checklist.

	Criterion															Total	
	Study quality					Study reporting											
Study	Eligibility criteria	Randomization specified	Allocation concealment	Similar groups at baseline	Blinding of assessor	Study reporting			Intention-to-treat analysis	Between-group statistical comparisons reported		Point and variability measures	Activity monitoring in control groups	Relative exercise intensity remained constant	Exercise volume and energy expenditure	Scores	Rating quality
						Participants adherence > 85%	Adverse events	Exercise adherence		primary outcome	secondary outcome						
	1	2	3	4	5	6a	6b	6c	7	8a	8b	9	10	11	12		
Beck & Norling (2010)	1	1	1	1	0	1	1	0	1	1	1	1	1	1	1	13	Excellent
Camacho-Cardenosa et al. (2019)	1	1	1	1	1	1	1	1	1	1	1	1	0	1	1	14	Excellent
Cheng et al. (2021)	1	0	1	1	1	0	0	0	1	1	0	1	1	1	1	10	Good
Santin-Medeiros et al. (2015)	1	1	1	1	0	0	1	0	1	1	1	1	1	1	1	12	Good
Song & Yang (2021)	1	1	0	1	1	1	0	0	1	1	0	1	1	1	1	11	Good
Verschueren et al. (2004)	1	1	1	1	1	1	0	0	1	1	1	1	1	1	1	13	Excellent
Zha et al. (2012)	1	1	1	1	1	1	1	1	1	1	1	1	1	1	1	15	Excellent

Additionally, two authors (DAM and AGM) assessed the risk of bias using the second version of the Cochrane risk-of-bias tool for non-randomized studies (RoB 2) (Sterne et al., 2019) in the following domains: (i) risk of bias arising from the randomisation process; (ii) risk of bias due to deviations from intended interventions; (iii) risk of bias due to missing outcome data; (iv) risk of bias in measurement of the outcome; and (v) risk of bias in selection of the reported result. Studies were categorized as having a low risk of bias if they received a “low risk” rating across all domains. Studies were considered to have some concerns of bias if at least one domain received a “some concerns” rating, a high risk of bias if at least one domain was rated as “high risk,” or if there were multiple domains rated as “some concerns” that may affect the validity of the results. Weighted summary and traffic light risk-of-bias plots for randomized included studies were produced using the risk-of-bias visualization (robvis) online tool (https://mcguinlu.shinyapps.io/robvis/) (McGuinness & Higgins, 2021). Discrepancies were resolved through discussion with another author (DMPF).

### Certainty of evidence

The authors DAM and TAFA determined the certainty of evidence following the GRADE (Grading of Recommendations, Assessment, Development and Evaluation) scale (Guyatt et al., 2008), of which procedures are available online at https://www.gradepro.org/ (McMaster University and Evidence Prime Inc., Hamilton, Canada). This scale is based on four categories of recommendation for grading the results of the studies according to the following levels: (i) high (*i.e*., results from future researches shall not alter the confidence on previous findings or estimates); (ii) moderate (*i.e*., results from future researches might probably alter the confidence on previous findings or estimates); (iii) low (*i.e*., results from future researches will probably alter the confidence on previous findings or estimates); and (iv) very low (*i.e*., previous findings or estimates have high uncertainty) (Guyatt et al., 2008).

Since the current values of WBV training effects on aBMD were selected from randomized controlled trial studies, the certainty of evidence is *a priori* considered high. However, this *a priori* grade can be reduced when accounting for: (1) the risk of bias measured with the TESTEX checklist (score < 6), and Cochrane’s risk of bias tool for randomized studies (RoB 2) (high bias in any domain); (2) the inconsistency measured by visual inspection of the effect size estimate, confidence interval overlap, and low (heterogeneity ≤ 25%) or significative (*p* < 0.05) heterogeneity; (3) the indirectness by analysing the alignment between the selected studies and current study goals; (4) the imprecision determined by sample power value (*e.g*., β < 0.80) in association with either the probability of Type II error (*i.e*., fail to reject the hypothesis that the effect of WBV training on BMD is null) or null Hedge’s g (≤0.0); and (5) publications bias due to the reduced number of studies (*n* < 10). Discrepancies were analysed by another author (DMPF).

### Statistical analysis

The statistical analysis was performed by an author (DAM) and reviewed by a second (ABP). The magnitude of the study results was determined by Hedges’ *g* and a 95% confidence interval (CI_95%_). For these estimates, the sample size, mean values, and standard deviation of BMD pre- and post-training for each anatomical site in the femur, spine, and hip in each condition (applied WBV training protocols) of each study included in the meta-analysis were used. The relative effects of training (∆%) were calculated in percentages according to Eq. (1) (Massini et al., 2022).

(1) $$\Delta {\rm \%} = \; \left[ {\displaystyle{{\left( {{\bar x}{_{post}}- {\bar x}{_{pre}}} \right)} \over {{\bar x}{_{pre}}}}} \right]\cdot 100$$

where “Δ%” is the training effect in percentage, “ $\bar{\rm  x}_{\rm pre}$” is the mean BMD before training, and “ $\bar{\rm  x}_{\rm post}$” is the mean BMD after training. The study estimates were combined in the meta-analysis using a random-effects model and presented as forest plots. Inconsistency was checked using the results of the meta-analysis, based on visual inspection of Hedges’ *g* estimates with overlapping or non-overlapping CI_95%_, as well as statistical tests for heterogeneity (*I*^2^) determined by combining the Cochran Q test (α < 0.10) with the Higgins test (Higgins & Thompson, 2002). Heterogeneity (*I*^2^) was categorized as follows: 0% < *I*^2^ ≤ 25% (no heterogeneity), 25% < *I*^2^ ≤ 50% (low heterogeneity), 50% < *I*^2^ ≤ 75% (moderate heterogeneity), and >75% (high heterogeneity) among studies (Massini et al., 2022). Sensitivity analysis to identify potentially influential or outlier WBV training protocols was performed using the amplitude defined as 1.5 times the interquartile range (IQR = Q3 − Q1) for Hedges’ *g*. If one or more studies were identified as outliers, the overall analysis was performed after removing the study(ies). Publication bias analyses were not assessed due to the inclusion of fewer than 10 studies (Higgins, Thomas & Chandler, 2023). The effect size for Hedges’ *g* was categorized as ≤0.19 (trivial), 0.20–0.59 (small), 0.60–1.19 (moderate), and ≥1.20 (large) (Hopkins et al., 2009). A significance level of α = 0.05 was adopted for all statistical procedures.

## Results

**Figure 1:**
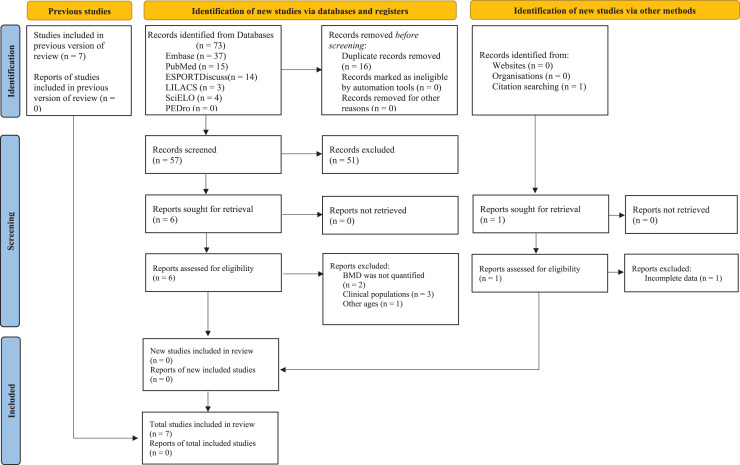
Whole-body vibration PRISMA flow diagram.

Figure 1 presents the flowchart for all stages of the systematic review and meta-analysis, and Table 2 outlines the key characteristics of the seven included studies; three were conducted in Europe (Verschueren et al., 2004; Santin-Medeiros et al., 2015; Camacho-Cardenosa et al., 2019;), three in Asia (Zha et al., 2012; Cheng et al., 2021; Song & Yang, 2021), and one in Oceania (Beck & Norling, 2010). These studies included 202 participants (~94% women and ~6% men) aged from 55 to 93. Regarding the twelve included training protocols, five different vibration platforms were observed. Training protocol variables included daily sessions lasting between 6 and 30 min, frequencies ranging from 12.5 to 55 Hz, intensity (acceleration) between 0.30 and 5.09 g, and amplitude between 0 and 14 mm. Weekly frequencies ranged from two to three times, and the protocols had durations between 18 and 32 weeks. Finally, a relative aBMD variation was observed for anatomical sites of the femoral neck ( $\Delta$% = 1.3 ± 2.3, CI_95%_ [−0.3% to 2.6%]), total femur ( $\Delta$% = 4.1 ± 5.2, CI_95%_ [0.8–7.3%]), and lumbar spine ( $\Delta$% = 1.6 ± 2.8, CI_95%_ [−0.5% to 3.6%]).

**Table 2:** Main characteristics of the selected studies regarding population features, WBV protocol, and effects on BMD.

Study	Participants					Whole-body vibration protocol						Areal bone mineral density		
	Groups	*n* Sex	Age (years) Height (cm) Weight (kg)	Instrument	Session	Frequency	Intensity (accelerations)	Amplitudes	Frequency	Study duration	Bone sites	Pre-training	Post-training	Δ%
					(Duration)	(Hz)	(g)	(mm)	(times week)	(weeks)		(g/cm^2^)	(g/cm^2^)	
Beck & Norling (2010)	WBV_HI_	15	68.9 ± 7.0	Galileo 2000, Germany	2 × 3 min, 1 min rest (Total 6 min)	30	1.0	0–14	2	32	Fn	0.750 ± 0.112	0.741 ± 0.114	−1.20
		Women	157.1 ± 6.0								Tr	0.591 ± 0.120	0.605 ± 0.112	2.37
			61.4 ± 8.9								LS	0.876 ± 0.122	0.872 ± 0.120	−0.46
	WBV_LI_	13	68.5 ± 8.6		15 min	30	0.3	–			Fn	0.749 ± 0.156	0.739 ± 0.156	−1.34
		Women	160.2 ± 7.0								Tr	0.591 ± 0.127	0.577 ± 0.137	−2.37
			68.4 ± 10.3								LS	0.941 ± 0.200	0.941 ± 0.206	0.00
Camacho-Cardenosa et al. (2019)	WBV	21	69.0	Galileo 2000, Germany	4 × 0.5 min, 1 min rest.	2.6	2.55	14	2	18	InTr	1.048 ± 0.351	1.074 ± 0.347	2.48
		Women	–											
		6	–											
		Men												
Cheng et al. (2021)	WBV_MFr_	19	64.8 ± 3.8	American-made powerplate vibrometers	20 min	20	–	3.0	3	24	Fn	0.790 ± 0.080	0.810 ± 0.090	2.53
		Women	158.2 ± 7.5								Tr	0.660 ± 0.110	0.710 ± 0.080	7.58
			59.3 ± 7.2											
	WBV_HFr_	18	65.1 ± 3.2		20 min	40	–	3.0	3	24	Fn	0.790 ± 0.100	0.820 ± 0.070	3.80
		Women	157.7 ± 6.0								Tr	0.660 ± 0.060	0.730 ± 0.100	10.6
			58.5 ± 7.3											
Santin-Medeiros et al. (2015)	WBV	19	82.3 ± 5.1	Fitvibe Excel Pro, Bilzen, Belgium	20 min	20	–	2.0	2	32	Fn	0.620 ± 0.090	0.610 ± 0.080	−1.75
		Women	–								Tr	0.570 ± 0.090	0.560 ± 0.090	−2.88
			–											
Song & Yang (2021)	WBV_LA_	19	63.9 ± 2.1	Power Plate vibrator system (Performance Health Systems, Northbrook, IL, USA)	20 min	45	–	2.0	3	24	Fn	0.800 ± 0.070	0.800 ± 0.050	0.00
		Women	158.8 ± 4.6								Tr	0.660 ± 0.030	0.660 ± 0.050	0.00
			57.3 ± 3.3								L_2–4_	0.970 ± 0.080	0.990 ± 0.110	2.06
	WBV_MA_	18	64.1 ± 1.7		20 min	45	–	3.0	3	24	Fn	0.790 ± 0.040	0.800 ± 0.030	1.27
		Women	158.5 ± 5.2								Tr	0.670 ± 0.110	0.740 ± 0.060	10.4
			57.2 ± 4.6								L_2–4_	0.960 ± 0.090	0.960 ± 0.100	0.00
	WBV_HA_	19	64.2 ± 1.8		20 min	45	–	4.0	3	24	Fn	0.790 ± 0.030	0.820 ± 0.060	3.80
		Women	159.1 ± 6.6								Tr	0.660 ± 0.090	0.730 ± 0.110	10.6
			56.9 ± 4.1								L_2–4_	0.970 ± 0.120	1.040 ± 0.080	7.21
Verschueren et al. (2004)	WBV	15	64.6 ± 3.3	PowerPlate, Amsterdam. The Netherlands	30 min	35–40	2.28–5.09	1.7–2.5	3	24	Fp	0.878 ± 0.136	0.886 ± 0.134	0.91
		Women	159.0 ± 5.0								L_1–4_	0.904 ± 0.143	0.901 ± 0.145	−0.33
			65.5 ± 8.9											
Zha et al. (2012)	WBV	21	77.7 ± 7.8	MYF Testing Equipment, Guangzhou, China.	1 × 10 min 1 min rest. 1 × 5 min 1 min rest. 1 × 5 min 1 min rest. (Total 6 min)	Vertical vibration changed cyclically between 45 and 55 Hz, at 1 Hz per second.	0.3	8	3	24	Fn	0.589 ± 0.121	0.608 ± 0.121	3.22
		Women	154.0 ± 8.0								LS	0.751 ± 0.146	0.770 ± 0.146	2.52
		6	54.4 ± 11.5				0.5							
		Men												
							0.8							

**Note:**

Fn, Femoral neck; Fp, total proximal femur; InTr, Intertrochanter; L, lumbar vertebrae; LS, lumbar spine; n, number of participants; Tr, trochanter; WBV, whole-body vibration.

### Meta-analysis

**Figure 2:**
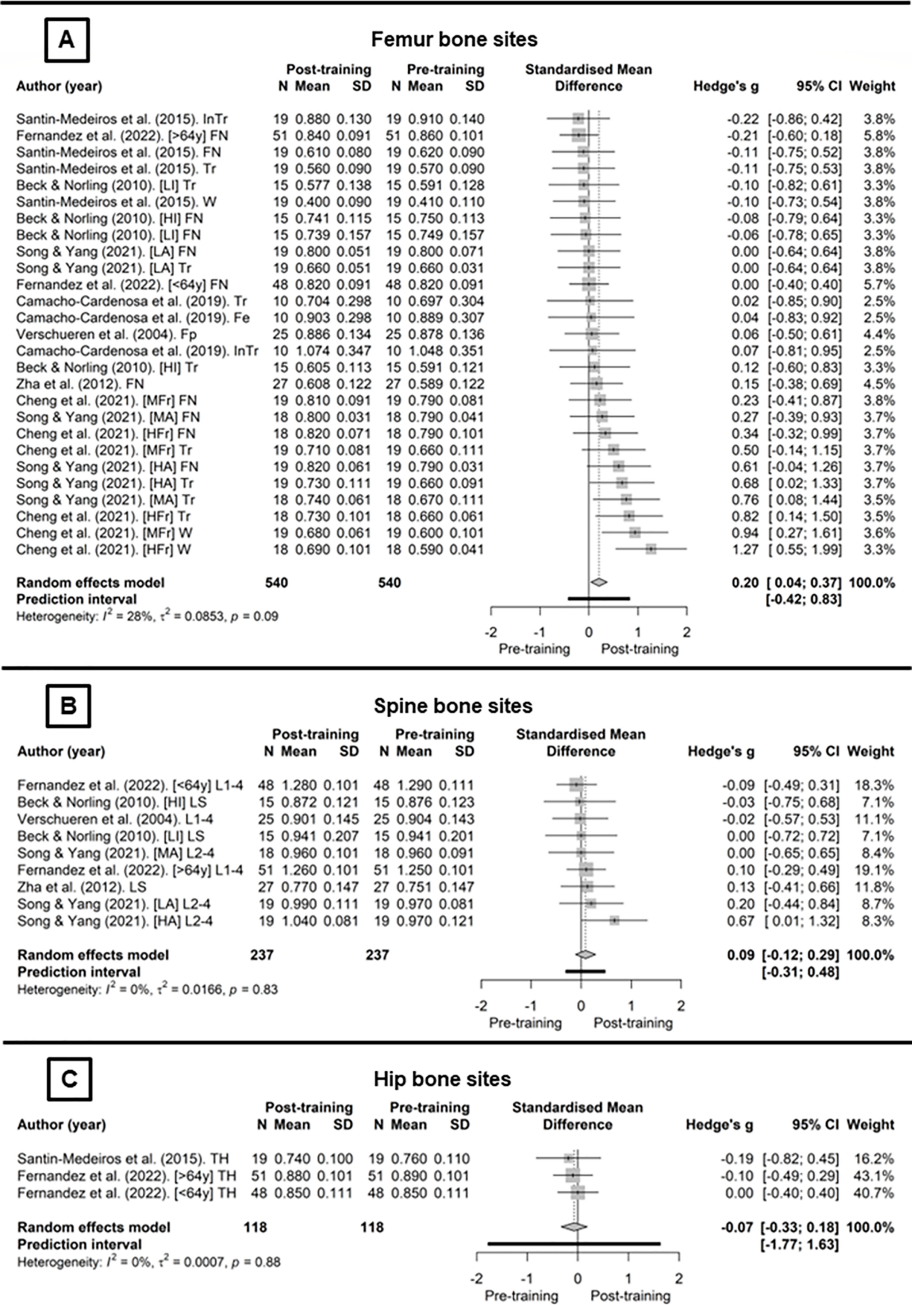
Whole-body vibration meta-analysis (Santin-Medeiros et al., 2015; Fernandez et al., 2022; Beck & Norling, 2010; Song & Yang, 2021; Camacho-Cardenosa et al., 2019; Verschueren et al., 2004; Zha et al., 2012; Cheng et al., 2021).

Figure 2 presents the WBV training protocols for the body regions included in the meta-analysis. The studies that analyse the femoral neck (Fig. 2A) combined with the random-effects model showed no significant effect of WBV protocols (*g* = 0.15, CI_95%_ [−0.08 to 0.39], *p* = 0.19, (trivial)). Inconsistency analysis through visual inspection of the overlap of CI_95%_ combined with statistical tests showed no heterogeneity (*I*^2^ = 0%; Q_[8]_ = 3.98, *p* = 0.86). For the total femur (Fig. 2B), using the random-effect model with no heterogeneity (*I*^2^ = 14.4%; Q_[9]_ = 10.51, *p* = 0.31), there was significant effect of the WBV protocols on BMD (*g* = 0.28, CI_95%_ [0.01–0.], *p* = 0., (small)). For the lumbar spine (Fig. 2C), the random-effect model showed no heterogeneity (*I*^2^ = 0%; Q_[6]_ = 3.37, *p* = 0.76), and also no significant effect of WBV protocols on BMD (*g* = 0.13, CI_95%_ [−0.13 to 0.39], *p* = 0.31, (trivial)).

### Methodological quality, risk of bias and certainty of evidence

Table 1 presents the results of each TESTEX methodological quality scale criterion for all included studies. Four studies showed excellent methodological quality (Verschueren et al., 2004; Beck & Norling, 2010; Zha et al., 2012; Camacho-Cardenosa et al., 2019) and three were rated as good (Santin-Medeiros et al., 2015; Cheng et al., 2021; Song & Yang, 2021). Therefore, the mean methodological quality value presented by the TESTEX checklist was 12.6 points (excellent quality), ranging between 10 and 15 points.

**Figure 3:**
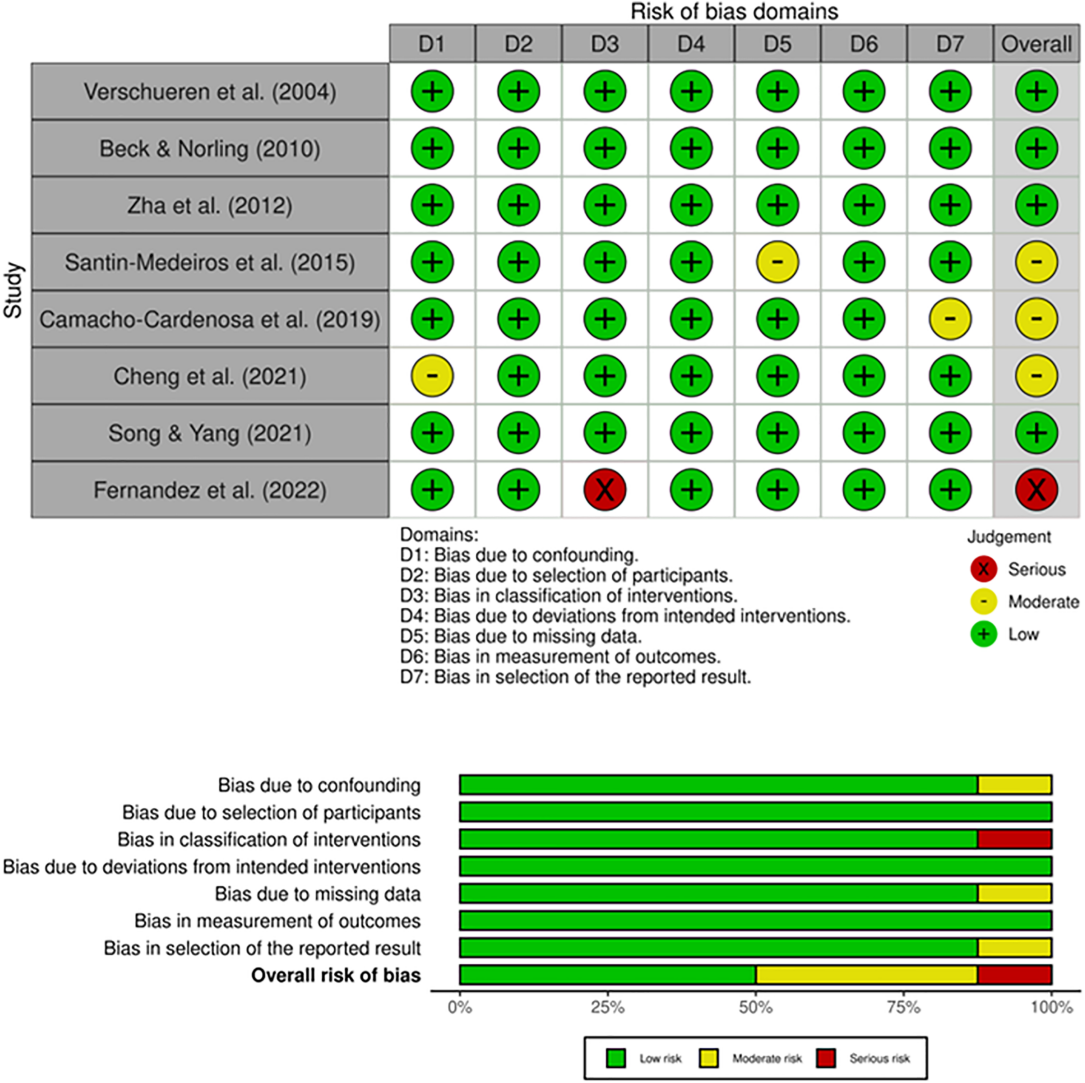
Whole-body vibration publications bias (Santin-Medeiros et al., 2015; Fernandez et al., 2022; Beck & Norling, 2010; Song & Yang, 2021; Camacho-Cardenosa et al., 2019; Verschueren et al., 2004; Zha et al., 2012; Cheng et al., 2021).

Regarding the risk of bias presented in the upper panel (Traffic light plot) of Fig. 3, some concerns were observed in four studies (Cheng et al., 2021; Santin-Medeiros et al., 2015; Song & Yang, 2021; Zha et al., 2012) related to bias arising from the randomisation process. The overall risk of bias presented in the lower panel (weighted bar plot) of Fig. 3 showed 43% low risk, and 57% some concerns.

The GRADE analysis is shown in Table 3. According to the GRADE scale, the current results were supported by evidence ranging from low to high certainty levels, which might be considered a concern since the interventions have hypothesized improvements in health and quality of life of the participants.

**Table 3:** GRADE assessment for the certainty of evidence.

Certainty assessment								No of patients		Effect		Certainty	Importance
Outcomes	No of studies	Study design	Risk of bias	Inconsistency	Indirectness	Imprecision	Publications bias	Pre-training	Post-training	Hedges’ *g* (95% CI)	MD (95% CI)		
Femoral neck aBMD	5	Randomised trials	Not serious	Not serious	Not serious	Very serious^a^	Not assessed	169	169	0.15 [−0.08 to 0.39]	0.01 [0.00–0.03]	⨁⨁◯◯ Low^a^	CRITICAL
Total femur aBMD	6	Randomised trials	Not serious	Not serious	Not serious	Not serious	Not assessed	177	177	0.28 [0.01–0.55]	0.03 [0.01–0.05]	⨁⨁⨁⨁ High	CRITICAL
Lumbar spine aBMD	4	Randomised trials	Not serious	Not serious	Not serious	Very serious^b^	Not assessed	138	138	0.13 [−0.13 to 0.39]	0.02 [−0.01 to 0.05]	⨁⨁◯◯ Low^b^	CRITICAL

**Notes:**

CI, Confidence interval.

MD, Mean differences (g/cm^2^).

a The sample size is small (β = 0.49) and there is a null effect (95% CI [−0.08 to 0.39]) in the Femoral neck of Whole-Body Vibration training in older adults.

b The sample size is small (β = 0.33) and there is a null effect (95% CI [−0.13 to 0.39]) in Lumbar Spine of Whole-Body Vibration training in older adults.

### Sensitivity analysis

The examination of outliers (1.5∙IQR) of Hedges’ *g* revealed one WBV protocol for the lumbar spine region (Song & Yang (2021) (HA) L_2–4_). However, the study by Song & Yang (2021) did exhibit one bias considered a concern. After removing the protocol from Song & Yang (2021) (HA) (L_2–4_), there was no change in heterogeneity (*I*^2^ = 0%; Q_[5]_ = 0.45, *p* = 0.99), although the overall analysis reduced (*g* = 0.05, CI_95%_ [−0.20 to 0.31], *p* = 0.68, (trivial)), and $\Delta$% also decreased to 0.6 ± 1.3 (CI_95%_ [−0.4% to 1.7%]).

## Discussion

The primary objective of this systematic review was to investigate the effect of WBV training *per se* on aBMD in older adults. The current meta-analysis evidenced with low certainty of evidence a significant effect of WBV training on anatomical sites in the total femur region throughout 18 to 32 weeks. However, this effect showed no statistical significance for the femoral neck and lumbar spine regions, indicating that WBV training had specific effects on different body regions.

Only two studies included in this systematic review included male participants (Zha et al., 2012; Camacho-Cardenosa et al., 2019). The low percentage of male participants and the lack of separate analyses by sexes do not allow applying the results to men or comparing them with women (Massini et al., 2022). The anthropometric characteristics (height and body mass) were similar among the included studies, although age showed a wide range (approximately 40 years). The limited number of studies included prevented an assessment of whether the effect of manipulating the variables in the WBV training is altered with age during subgroup analyses (Abazović, Paušić & Kovačević, 2015).

Variables of effective WBV training are not yet fully defined (Slatkovska et al., 2010; Santin-Medeiros et al., 2015; Wuestefeld et al., 2020; van Heuvelen et al., 2021), and the way that previous studies followed planning the variables in WBV training are partially aligned with each other, therefore making it difficult to observe a standardized recommendation in the literature (Abazović, Paušić & Kovačević, 2015; Marín-Cascales et al., 2018; de Oliveira et al., 2023). Regarding the recommendation for the variable frequency, only the study by Zha et al. (2012) used a frequency of 55 Hz. However, frequencies above ~50 Hz should be used with caution since an adverse events such as acute muscle pain and even hematoma might occur in untrained individuals (Abazović, Paušić & Kovačević, 2015; Marín-Cascales et al., 2018). As for the variable amplitude, investigations used both lower (Beck & Norling, 2010; Fernandez et al., 2022), and higher oscillations (Beck & Norling, 2010; Zha et al., 2012; Camacho-Cardenosa et al., 2019). The difference in the effect ( $\Delta$%) can be explained by the combination with the rate of sessions per week, where studies with lower oscillations and two weekly sessions (Beck & Norling, 2010; Santin-Medeiros et al., 2015) had a smaller $\Delta$% compared to the study with three weekly sessions (Zha et al., 2012).

In contrast, when the number of sessions per week remain unchanged the effect of WBV training (in ∆%) can be enhanced by modifying (increasing) the amplitude of the frequency, but this planning strategy has shown positive results mainly in protocols previously designed with high amplitudes (Song & Yang, 2021). Therefore, the most significant effects were reported for protocols with frequencies between 40 and 45 Hz, amplitudes between 3.0 and 4.0 mm, and three sessions per week, as seen in the studies by Cheng et al. (2021) and Song & Yang (2021). Although the studies reported no adverse events during WBV training combining high accelerations, the safety of this procedure should be carefully considered before designing training for individuals showing degrees of musculoskeletal systems frailty. Therefore, is also recommended to consider that the increase in the amplitude of the vibration frequency (*i.e*., >0.5 mm) can produce higher peak acceleration of the platform at lower frequencies (*i.e*., <50 Hz) than low amplitude (*i.e*., 0.1 to 0.01 mm), and since the acceleration and frequency relate to each other in an non-linear order with a tendency to reach a plateau, the transmissibility of vibration-induced acceleration from platform to the body attenuates more with high-amplitude low-frequency stimuli, and at high frequencies for low amplitudes. This attenuation is therefore important to prevent tissue damage since body acceleration might not corresponded to platform acceleration, hence exceeding a safety range of stimuli (Kiiski et al., 2008). Regarding the duration of the studies, this did not limit the presented results because bone formation and stabilization occur between 3–4 and 6–8 months, respectively (Kohrt et al., 2004; Massini et al., 2022). Only the study of Camacho-Cardenosa et al. (2019) had a duration of 4.2 months, although the reports for improvements in aBMD were aligned with other studies (Arce-Esquivel & Ballard, 2015; Massini et al., 2022) regardless of the applied training modality.

The main constraint in conducting meta-analyses and establishing robust evidence on the effects of WBV training on aBMD is the heterogeneity present in the methodologies of the studies (Fischer et al., 2019). In this regard, considering only the intragroup effect (pre- *vs*. post-training) is a strategy to eliminate the confounding bias related to the different control models in the included studies (Dolan et al., 2020; Massini et al., 2022). However, the heterogeneity absence in this meta-analysis is due to the differences in WBV training protocols (*e.g*., intervention duration, frequency and volume of sessions, type, and amplitude of mechanical vibrations and exercises performed on the platform) (Fischer et al., 2019).

Therefore, the small effect size for the total femur region and the trivial effects for the femoral neck and lumbar spine regions (Hopkins et al., 2009), with their respective relative gains ( $\Delta$%), demonstrate that aBMD response to WBV training is like resistance training (Arce-Esquivel & Ballard, 2015; Fischer et al., 2019; Massini et al., 2022). This suggests that bone tissue response to exercise could not be compared to other body tissues (*e.g*., muscle) (Massini et al., 2022), despite the substantial potential to adapt to exercise, according age, sex (related to body composition, *i.e*., difference in fat and muscle mass), and type of stimuli (impact or tensional, *i.e*. resistance exercise) (Guimarães et al., 2018; Massini et al., 2023). Thus, the differences in results between the total femur region and the femoral neck and lumbar spine could be explained by: (i) the mechanotransduction varying in different body regions due to the nonlinear musculoskeletal system and different body positions influencing the amount of stimuli each region receives during WBV training (Kiiski et al., 2008; Abazović, Paušić & Kovačević, 2015; Marín-Cascales et al., 2018); and (ii) the difference in the number of WBV training protocols (large variance and small sample size) included compared to the total femur region, reducing the statistical power to observe the effect (Slatkovska et al., 2010; Marín-Cascales et al., 2018; Santo André et al., 2023). In addition, aging might reduce the effect of WBV training on aBMD, since the mechanical stimulation from vibration cannot be effective in activating muscles adequately in older individuals, therefore also reducing the stimuli of muscles on bone mineral metabolism (Cheng et al., 2021; Song & Yang, 2021). Although information available about how different bone sites adjust mineral mass and density to WBV training in older adults requires more studies, the aging-related reduction of aBMD is a process that should be considered when analysing (any) exercise intervention effectiveness on aBMD, since the smallest increase when reduction is expected is clinically relevant. For example, a reduction of 2.13% of femoral neck aBMD was observed between healthy older women (age 57.33) after 12 months of non-training involvement, while those who underwent training for similar period improved by 0.04%, which means a possible effect of 2.17% (Rubin et al., 2004).

Regarding the methodological quality (TESTEX = 12.6) of this systematic review (Smart et al., 2015), its results were based on studies with good methodological quality (Nunes et al., 2020). However, in some cases the TESTEX score revealed limitations in reporting the exercise protocol with adequate details. Nevertheless, this is essential for interpreting the results of the studies selected after the search screening (Fischer et al., 2023). Thus, the TESTEX results were compared with the risk of bias (Sterne et al., 2019), and from the risk of bias analysis it was observed that the studies by Cheng et al. (2021), Santin-Medeiros et al. (2015), Song & Yang (2021), and Zha et al. (2012) did not report details of the process of group formation and randomization. Although the groups did not present differences between them at the beginning of the study, this may have influenced the results, as suggested by Sterne et al. (2019).

The analysis of the GRADE scale indicated a low level of certainty of evidence for the results of the revised studies, which is aligned with the findings from these studies, suggesting the importance of new studies for strengthening the level of evidence on the effect of WBV training on aBMD, as verified by the current metanalysis. The reduction of the level of certainty of evidence on the results from the randomized clinical trials was related to the criteria of imprecision being enhanced to the reduced sample power. Therefore, factors such as the reduced number of qualified studies, low sample size, and the wide range of 95% confidence interval contributed to the low certainty of evidence, supporting previous discussions on the factors contributing to the limitation of evidence (Guyatt et al., 2008; Massini et al., 2022), as well as constraining the inferences from the unrestrictive effects of WBV training for aBMD health in an elderly population.

The sensitivity analysis (Viechtbauer & Cheung, 2010) identified only the protocol from the study by Song & Yang (2021) (HA)_L2–4_ as an outlier, although the other protocol from this same study (WBV_MA_), as well as one other protocol (WBV_HFr_) from the study by Cheng et al. (2021) showed much higher effects (>10%) compared with other studies, regardless of the exercise planned for the training protocol (*i.e*., aerobic, resistance, impact) (Liu, Brummel-Smith & Ilich, 2011; Arce-Esquivel & Ballard, 2015; Beavers et al., 2017; Mohammad Rahimi et al., 2020; Massini et al., 2022). One possible explanation for the protocols of these studies showing higher effects (Cheng et al., 2021; Song & Yang, 2021) is that they combined frequencies (40 and 45 Hz) with amplitudes (3.0 and 4.0 mm), while other studies prioritized only frequency (Verschueren et al., 2004; Fernandez et al., 2022). The study of Zha et al. (2012), using higher frequencies and amplitudes (45 to 55 Hz and 8.0 mm), had good results (femoral neck = 3.2% and lumbar spine = 2.5%), although less pronounced, which may have occurred due to excessive stimulation (*i.e*., the negative influence of the high overload) (Abazović, Paušić & Kovačević, 2015; Marín-Cascales et al., 2018). However, this is an observation from the analysis of the previous studies, and therefore this is a supposition that should be still verified by new clinical studies (Marín-Cascales et al., 2018; Harijanto et al., 2022).

## Limitation

A limitation of this systematic review is the small number of included studies, which hinders the power of statistical tests to detect smaller effects (in femoral neck and lumbar spine). It also limits subgroup analyses and explores the potential moderators in meta-regression (Abazović, Paušić & Kovačević, 2015; Mohammad Rahimi et al., 2020; Massini et al., 2022; Santo André et al., 2023). Additionally, the small number of male participants prevents the application of these results to this population, even though men can also develop osteopenia and osteoporosis due to factors such as physical inactivity and being bedridden for prolonged periods (Slatkovska et al., 2010; Ireland, Rittweger & Degens, 2014; Abazović, Paušić & Kovačević, 2015). Generalizing the results should be seen with caution due to potential limitations in the heterogeneity of WBV planning (Slatkovska et al., 2010; Moreira-Marconi et al., 2016), as well as due to the low level of certainty of evidence for the results of WBV training as suggested by GRADE analysis.

Therefore, future studies should consider the inclusion of men, as well as a comparison between sexes, in order to avoid sex as a confounding factor (Massini et al., 2022). Regarding the differences between WBV training, it is also suggested to analyse in future studies the combination of frequencies between 40 and 45 Hz, in amplitudes between 3.0 and 4.0 mm, and with at least three training sessions per week, as recommended by Cheng et al. (2021) and Song & Yang (2021). Such future studies must also attempt to plan protocols with intervention for a minimum period of six months to be aligned with the response time of bone remodelling (Kohrt et al., 2004; Massini et al., 2022). However, future studies must report information about commercial devices (brand name and type), type of vibration (synchronous, side-alternating), vibration frequency (or frequencies in Hz), peak-to-peak displacement of the vibration in mm (for side-alternating vibration, give this information for a specific landmark, *e.g*., the second toe), intensity (preferably in multiples of gravity acceleration), accuracy of vibration parameters (preferably based on the reference to literature), changes of the vibration settings during the study, and rationale for choosing specific vibration settings (in accordance on literature) (Rauch et al., 2010; van Heuvelen et al., 2021). The studies should also report support devices for the participants during training intervention (*e.g*., none when standing freely, holding on to a railing), type of footwear (*e.g*., shoes, socks, or barefoot), the posture of the participant when standing on the plate (*e.g*., knee and hip angle, standing on one or two legs, leaning on toes or heels, trunk upright or tilted forward), and describe exercise performed on the plate (*e.g*., static or dynamic exercises) (Rauch et al., 2010; van Heuvelen et al., 2021). Additionally, some studies recommend monitoring the occurrence of adverse events, such as acute muscle pain (Kiiski et al., 2008; Abazović, Paušić & Kovačević, 2015; Marín-Cascales et al., 2018; Moreira-Marconi et al., 2019; de Oliveira et al., 2023).

## Conclusions

The reviewed studies indicated that WBV training had a statistically significant but clinically small effect only on the total femur, with no significant effects observed in the femoral neck and lumbar spine, demonstrating different effects on each body region (mainly for women). However, the current analysis of the literature reinforces that WBV training is a safe and effective non-pharmacological intervention for improving bone mass and density, particularly in the femur region. Nevertheless, a detailed analysis of the effects of WBV training on aBMD still requires appropriate and controlled variables of training to ensure ecological validity, and application as an effective clinical practice in improving bone health. Therefore, WBV is gaining popularity as a treatment tool to improve musculoskeletal disorders and improve health-related quality of life.

## Supplemental Information

10.7717/peerj.19230/supp-1 Supplemental Information 1 PRISMA checklist.

10.7717/peerj.19230/supp-2 Supplemental Information 2 Rationale and Contribution.

## Additional Information and Declarations

### Data Availability

The following information was supplied regarding data availability:

This is a systematic review/meta-analysis.
