# Supplementary material for: Effect of whole-body vibration training on bone mineral density in older adults: a systematic review and meta-analysis
Source: PeerJ. 2025 May 16;13:e19230. doi: 10.7717/peerj.19230 (PMC12087579; doi:10.7717/peerj.19230)
Supplement: Supplemental Information 2 [file peerj-13-19230-s002.docx]

Supplemental File with the following information:

**1. The rationale for conducting the meta-analysis;**

This systematic review (PROSPERO – CRD 42023395390) analyzed the effects of whole-body vibration (WBV) training on bone mineral density (BMD) at anatomical sites most affected by osteoporotic fractures in older adults. In this systematic review all type of studies (regardless of whether they are randomized or non-randomized) were included, but the measurements with DXA technology was mandatory. Therefore, the current study provided standardized information of BMD adjustments with WBV intervention, thus enabling high reproducibility, and confident analysis of WBV effects. In addition, age and training duration were controlled, since age is known to influence BMD the regression analysis was conducted to examine the effect of WBV on age-group experiencing strong resistance to BMD alterations due to the age, as well as the analysis considered the training duration to avoid findings of WBV effect with influence of time. Finally, the results (when positive, negative, or null) indeed demonstrated the protocol planning affecting BMD effectively.

**2. The contribution that the meta-analysis makes to knowledge in light of previously published related reports, including other meta-analyses and systematic reviews.**

From the literature survey were screened almost 614 records, from which were selected eight studies involving 301 participants were included, with methodological quality (TESTEX: 12.5, excellent quality) and risk of bias (50 % low, 37.5 % moderate, and 12.5 % serious risk). These studies demonstrating a significant effect of WBV training on femur BMD, with no significant effects observed in the spine and hip regions. In addition, the meta-regression analysis indicated that WBV training, regardless of duration, cannot reverse age-related bone loss. Therefore, the current literature analysis reinforces that WBV training is a safe and interesting alternative to avoiding bone mass and density critical reduction with aging, particularly in the femur region. However, it is necessary to highlight that in most of the studies analyzed the population sample was women, limiting the generalization of the present results to men and highlighting the importance of studies with the male population. Moreover, it is also important to note that the current study verify the effect of WBV training on BMD with no influence of confounding factors, unlike previous studies in which population was analyzed with no control for the use of dietary supplements (i.e. vitamins and minerals) (Slatkovska et al. 2010; Dionello et al., 2016; Marín-Cascales et al., 2018) and/or osteogenic drugs (i.e. medications and hormones) (Harijanto et al., 2021)

Therefore, WBV is gaining popularity as an effective clinical practice in improving bone health, and hence the current findings strengthen the WBV training as a treatment protocol to improve bone disorders and improve health-related quality of life, which can be useful for various healthcare professionals dealing with the elderly population.
